# Dependence on zopiclone: a case report

**DOI:** 10.3389/fpsyt.2025.1592065

**Published:** 2025-05-23

**Authors:** Nana Guo, Xuhui Zhou

**Affiliations:** ^1^ School of Clinical Medicine, Hunan University of Chinese Medicine, Changsha, Hunan, China; ^2^ Department of Addiction Medicine, Hunan Institute of Mental Health, The Second People’s Hospital of Hunan Province (Brain Hospital of Hunan Province), Changsha, Hunan, China

**Keywords:** zopiclone, addiction, dependence, withdrawal symptoms, case report

## Abstract

**Background:**

Zopiclone, a cyclopyrrolone derivative, is commonly employed for the short-term treatment of different types of insomnia. In comparison to benzodiazepines, Zopiclone appears to have a lower risk of abuse potential and withdrawal symptoms. Nevertheless, as the use of Zopiclone has increased, reports of abuse and dependence have begun to surface in the literature.

**Case presentation:**

This case report describes a 43-year-old male patient who has been taking zopiclone for 12 years, with a maximum daily dose of 112.5 mg. Two days after discontinuing the medication, the patient experienced withdrawal symptoms such as palpitations, sweating, irritability, hallucinations, and impulsive behavior, which resulted in his admission to a psychiatric hospital. After 17 days of treatment with diazepam, quetiapine fumarate, magnesium valproate, and trazodone hydrochloride, the patient’s withdrawal symptoms alleviated, sleep quality improved, and medication cravings decreased.

**Conclusion:**

Similar to benzodiazepines, Zopiclone may also result in abuse and dependency issues. Therefore, when prescribing Zopiclone, physicians must be fully aware of it.

## 1 Introduction

Insomnia is a condition defined by the perception of inadequate sleep, wherein patients commonly encounter difficulties in initiating sleep, sustaining sleep, or experiencing poor sleep quality (1). The prevalence of insomnia in the general population is estimated to vary between 5% and 50% (2). Epidemiological studies indicate that approximately one-third of adults report experiencing at least one symptom of insomnia (2). Insomnia poses not only a psychological burden for affected individuals but also represents a significant public health concern (3).

Zopiclone, which was approved in 1986 as the first non-benzodiazepine hypnotic in the European market, is typically classified with zaleplon and zolpidem as Z-drugs (4). These drugs produce hypnotic effects by reducing sleep onset latency and enhancing sleep quality and are primarily used for the short-term treatment of sleep disorders (5). Like benzodiazepines (BDZs), Z-drugs interact with the GABA_A_ receptor, enhancing GABA-mediated neuronal inhibition (6). Their higher affinity for the α1 subunit of the GABA_A_ receptor suggests a lower potential for abuse, tolerance, and withdrawal (6). These agents are characterized by rapid absorption and a short half-life, leading to minimal residual effects during the day (7). Consequently, the availability of Z-drugs has increased over time (8). Between 2008 and 2018, global consumption of Z-drugs increased at an annual rate of 3.28%, whereas benzodiazepine use declined (9). A study conducted at the McGill University Health Centre in Montreal, Canada, revealed a high prevalence of inappropriate Z-drug prescriptions, with 38% of inpatients receiving doses exceeding the recommended daily maximum. Additionally, 39% of prescriptions surpassed the advised duration of use (10). Growing evidence indicates that Z-drugs are associated with risks of abuse, dependence, and mortality (11).

This report presents a case of a 43-year-old male patient with a 12-year history of chronic zopiclone abuse, with a daily consumption of up to 112.5 mg. During withdrawal, the patient exhibited symptoms including palpitations, sweating, anxiety, irritability, hallucinations, and impulsive behavior. Detoxification was successfully managed through a comprehensive treatment approach that included diazepam tapering, antidepressants, antipsychotics, and mood stabilizers.

## 2 Case description

A 43-year-old male patient presented with insomnia that began after a romantic relationship breakdown in 2012, characterized by difficulty initiating sleep and, at times, a complete inability to sleep through the night. He was diagnosed with non-organic insomnia by an outpatient physician and was prescribed 7.5 mg of zopiclone nightly as a hypnotic agent. Despite this intervention, the patient reported dissatisfaction with the efficacy of the hypnotic medication and gradually increased the dose of the medication on his own, eventually reaching 37.5 mg per night, with still unsatisfactory sleep results. Inadequate nocturnal sleep resulted in daytime somnolence and irritability. During daytime naps, he found that zopiclone not only facilitated sleep but also induced relaxation and euphoria. The patient subsequently initiated covert consultations across various healthcare facilities, seeking prescriptions under the pretext of insomnia. The patient administered zopiclone at doses ranging from 15 to 22.5 mg, with a frequency of 2 to 3 times daily, particularly during periods of fatigue or distress. Upon cessation, he experienced withdrawal symptoms, including poor sleep, irritability, sweating, and dyspnea, which diminished upon resuming medication. This allowed him to maintain occupational functionality. By 2016, his zopiclone intake had escalated to 90 mg daily, resulting in sometimes memory impairment, dizziness, somnolence, and eventual unemployment. Attempts by an outpatient physician to substitute zopiclone with lorazepam were ineffective. In 2021, following zopiclone withdrawal, he exhibited irritability and auditory hallucinations, prompting a psychiatric hospital to prescribe olanzapine, valproate, and diazepam to manage his zopiclone use. However, this regimen was ineffective, and his zopiclone consumption increased to 112.5 mg daily. Recently, two days prior, he attempted withdrawal again, experiencing palpitations, sweating, tremors, anxiety, irritability, auditory hallucinations, and destructive behavior, which led him to an alcohol addiction center for inpatient treatment.

Regarding the patient’s medical history, there is no prior record of psychiatric disorders, nor is there any history of alcohol or psychoactive substance abuse. The patient has a smoking history of 22 years, with a daily consumption of 20 cigarettes. In terms of personal history, the patient exhibited normal growth and development, with average academic performance. Professionally, the patient worked as a driving instructor, demonstrating satisfactory job performance with minimal occupational stress, but later resigned due to zopiclone misuse. The patient is married, with a harmonious spousal relationship, and there are no significant findings in the family medical history.

Upon admission, the patient’s temperature was recorded at 36.8°C, with a respiratory rate of 30 breaths per minute, a pulse rate of 135 beats per minute, and blood pressure measuring 156/105 mmHg. Auscultation revealed clear breath sounds in both lungs, with no presence of dry or wet rales. The heart rate was regular at 135 beats per minute, with no pathological murmurs detected. Upon abdominal examination, no significant abnormalities were detected. Neurological assessment revealed tremors in both upper extremities. During the psychiatric evaluation, the patient was accompanied by family, demonstrating clear consciousness and accurate orientation. The patient was passively cooperative during interactions but exhibited poor concentration, auditory hallucinations, irritability, anxiety, and a craving for zopiclone. A preliminary assessment of cognitive function indicates that the patient’s general knowledge and comprehension judgment are intact, with memory and calculation abilities preserved. Insight remains present. The patient reported disrupted sleep-wake cycles and poor appetite. Routine blood, urine, and stool tests yielded normal results, as did blood glucose levels and liver and kidney function tests. Cardiac enzyme levels were within normal limits, and thyroid function tests were unremarkable. A urine drug screening indicates negative results for BDZs, morphine, ketamine, ecstasy, methamphetamine, and buprenorphine. The electrocardiogram showed sinus tachycardia, while both the electroencephalogram and head MRI indicated no significant abnormalities. The Hamilton Depression Rating Scale (HAMD) score is 17, the Hamilton Anxiety Rating Scale (HAMA) score is 26, and the Brief Psychiatric Rating Scale (BPRS) score is 38, indicating the presence of anxiety, depression, and psychotic symptoms in the patient.

The patient’s clinical presentation and history are consistent with disorders induced by sedatives, hypnotics, or anxiolytics, as described in the fifth edition of the Diagnostic and Statistical Manual of Mental Disorders (DSM-5).

Upon admission, the patient was administered oral diazepam at a dosage of 2.5 mg twice daily, alongside intravenous diazepam at 10 mg nightly to alleviate withdrawal symptoms. Additionally, sustained-release magnesium valproate was prescribed at 0.5 g daily to stabilize mood and reduce impulsivity. Quetiapine fumarate tablets were administered at 100 mg nightly to address psychotic symptoms and improve sleep. Trazodone hydrochloride was given at 50 mg daily to counter anxiety and depressive symptoms and enhance sleep quality. By the third day, the patient’s heart rate, blood pressure, and respiration normalized, though sleep disturbances persisted, with reported difficulty in falling asleep and slight improvement in anxiety, without significant impulsive behavior. The evening intravenous diazepam was transitioned to oral administration, with trazodone increased to 100 mg nightly and quetiapine to 200 mg. By the seventh day, sleep showed marginal improvement, auditory hallucinations resolved, and anxiety and depressive symptoms significantly improved (Hamd 13, Hama 18, BPRS 28), though a craving for zopiclone remained. The evening oral diazepam was reduced to 5 mg, trazodone increased to 150 mg, and quetiapine to 400 mg nightly. By the tenth day, anxiety and depressive symptoms continued to diminish, prompting a reduction in daytime oral diazepam to 2.5 mg. By the thirteenth day, withdrawal symptoms were nearly absent, with substantial improvement in anxiety and depression (Hamd 12, Hama 10), though sleep remained suboptimal, leading to the cessation of daytime diazepam and an increase in evening oral diazepam to 7.5 mg. Following medication adjustments, the patient reported improved sleep quality and opted for discharge four days later. At discharge, the patient exhibited no significant withdrawal symptoms, further improvement in anxiety and depression (Hamd 8, Hama 7), absence of psychotic symptoms, reduced craving for zopiclone, and improved sleep. No significant adverse drug reactions were observed during treatment. Post-discharge, medication will be gradually tapered in an outpatient setting based on the patient’s clinical status. Details of medications administered during hospitalization are shown in **Table 1**.

**Table 1:** Detailed medication administration.

Hospital days Medicine	Day 1	Day 2	Day 3	Day 4	Day 5
diazepam	10mg Qn IV+2.5mg Bid PO	10mg Qn IV+2.5mg Bid PO	10mg Qn PO+2.5mg Bid PO	10mg Qn PO+2.5mg Bid PO	10mg Qn PO+2.5mg Bid PO
Quetiapine	100mg Qn PO	100mg Qn PO	200mg Qn PO	200mg Qn PO	300mg Qn PO
Trazodone Hydrochloride	50mg Qn PO	50mg Qn PO	100mg Qn PO	100mg Qn PO	100mg Qn PO
Magnesium Valproate	0.25g Bid PO	0.25g Bid PO	0.25g Bid PO	0.25g Bid PO	0.25g Bid PO

Hospital days Medicine	Day 6	Day 7	Day 8	Day 9	Day 10
diazepam	10mg Qn PO+2.5mg Bid PO	5mg Qn PO+2.5mg Bid PO	5mg Qn PO+2.5mg Bid PO	5mg Qn PO+2.5mg Bid PO	5mg Qn PO+2.5mg Qd PO
Quetiapine	300mg Qn PO	400mg Qn PO	400mg Qn PO	400mg Qn PO	400mg Qn PO
Trazodone Hydrochloride	100mg Qn PO	150mg Qn PO	150mg Qn PO	150mg Qn PO	150mg Qn PO
Magnesium Valproate	0.25g Bid PO	0.25g Bid PO	0.25g Bid PO	0.25g Bid PO	0.25g Bid PO

Hospital days Medicine	Day 11	Day 12	Day 13	Day 14	Day 15
diazepam	5mg Qn PO+2.5mg Qd PO	5mg Qn PO+2.5mg Qd PO	7.5mg Qn PO	7.5mg Qn PO	7.5mg Qn PO
Quetiapine	400mg Qn PO	400mg Qn PO	400mg Qn PO	400mg Qn PO	400mg Qn PO
Trazodone Hydrochloride	150mg Qn PO	150mg Qn PO	150mg Qn PO	150mg Qn PO	150mg Qn PO
Magnesium Valproate	0.25g Bid PO	0.25g Bid PO	0.25g Bid PO	0.25g Bid PO	0.25g Bid PO

Hospital days Medicine	Day 16	Day 17			
diazepam	7.5mg Qn PO	7.5mg Qn PO			
Quetiapine	400mg Qn PO	400mg Qn PO			
Trazodone Hydrochloride	150mg Qn PO	150mg Qn PO			
Magnesium Valproate	0.25g Bid PO	0.25g Bid PO			

During a telephonic follow-up conducted two months post-discharge, the patient reported cessation of diazepam under outpatient physician supervision. However, the patient expressed dissatisfaction with sleep quality and exhibited a craving for zopiclone during periods of poor sleep. It has been recommended that the patient seek further treatment from a sleep specialist.

## 3 Discussion

Zopiclone is marketed as a safer alternative to BDZs because of its perceived safety profile, with earlier studies indicating a lower potential for abuse and dependency. A large-scale study in the UK involving 13,177 patients prescribed Zopiclone found no confirmed cases of dependence on the drug and also noted that overdoses were safe (12). Another researcher recognized the potential for Zopiclone abuse, especially among psychiatric patients, but reported only 22 instances of misuse, concluding that the incidence of abuse is negligible (13). A retrospective study indicated that tolerance, rebound, and withdrawal phenomena associated with Zopiclone are minimal (14).

Over time, reports of zopiclone abuse have emerged, particularly among individuals with alcohol dependence (15, 16), poly-substance abusers (17), or patients with other psychiatric disorders (18). In a clinic in central Dublin, the misuse of zopiclone is prevalent among clients participating in methadone maintenance treatment programs. Clients report that, while zopiclone does not induce the same degree of amnesia as BDZs, it enhances their heroin experience and promotes a desired sense of sedation and tranquility (17). A study investigating psychotropic drug dependence among alcohol-dependent patients in Sweden found that zopiclone dependence was significantly higher than that in the general population (15). Lam reported that the number of zopiclone abusers in a clinic serving drug abusers in Hong Kong, China, had increased significantly over the past four years, accounting for 30% of new cases. (19). This contrasts with our patient, who, aside from nicotine dependence, has no history of substance use or psychiatric disorders. The patient initiated the use of zopiclone primarily due to insomnia, and the absence of scientifically validated treatments for sleep disorders ultimately resulted in drug misuse and dependency. Consequently, chronic insomnia may represent a vulnerability factor for zopiclone abuse and dependence.

In our case study, the patient experienced relaxation and euphoria following the administration of zopiclone, indicating an anxiolytic effect and euphoria post-ingestion. This played a pivotal role in the patient’s drug dependency. This phenomenon contradicts the selective action of zopiclone on the GABA_A_ receptor α1 subunit. GABA_A_ receptor subtypes exhibit distinct regional and cellular distribution patterns in the brain (20). The α1 subunit receptors are distributed in most brain regions, particularly the cerebral cortex, and produce a hypnotic effect when activated. The α2 subunit receptor is located mainly in the hippocampus, amygdala, basal ganglia, and outer layers of the cerebral cortex, a region closely associated with the anxiolytic effects of BDZs. α3 subunit receptors are expressed in the thalamic reticular nucleus and the inner layers of the cerebral cortex, which have also been implicated in the anxiolytic effects of BDZs. α5 subunit receptors are predominantly found in the hippocampus and in the inner layers of the cerebral cortex, a region implicated in learning and memory (21). Unlike BDZs, which have a similar affinity for the α1, α2, α3, and α5 receptor subtypes, zopiclone has a higher affinity for GABA_A_ receptors containing the α1 subunit, resulting in a hypnotic effect but no anxiolytic effect (6). We hypothesize that at supra-therapeutic doses, zopiclone may lose its selectivity for the α1 subunit and interact with other subunits, such as α2 or α3, leading to anxiolytic, excitatory, and euphoric effects. Additionally, our subject and previous reports on zolpidem dependence (22) have noted memory impairment, potentially linked to α5 receptor activation. An earlier study also supports our results that zopiclone may not be selective in its action on GABA_A_ receptors (23). It is also possible that the anxiolytic and euphoric effects of zopiclone are related to α1 receptor activation. Given the complexity of GABA_A_ receptors and their functions (24), ongoing research is required, and future studies should further explore the interactions between zopiclone and different GABA_A_ receptor subunits.

To our knowledge, this is the second clinical case report documenting zolpidem misuse exceeding 100 mg per day. This patient was taking up to 112.5 mg of zopiclone per day. In addition to previously identified withdrawal symptoms such as insomnia, anxiety, tachycardia, and tremors indicating autonomic instability (25, 26), it is noteworthy that following the cessation of high-dose zopiclone, the patient experienced psychiatric abnormalities, including auditory anxiety, depression, auditory hallucinations, and impulsive behavior. Another report described a 74-year-old female who had been using high doses of zopiclone (112.5 mg/day) for over 20 years, along with diabetes, hypertension, and suspected ischemic heart disease, and who developed delirium upon withdrawal (27). Furthermore, two studies reported seizures resulting from high-dose zopiclone misuse. The first case involved a 36-year-old male who consumed 60–90 mg daily and had a history of depressive disorder and alcohol abuse, experiencing seizures after discontinuation (28). The second case involved a 76-year-old married woman with a history of depression who took 67.5 mg of zopiclone daily and experienced three typical seizures two days after abrupt withdrawal (29). Additionally, a report indicated that an 84-year-old female developed delirium after receiving a single dose of zopiclone (30). It is evident that withdrawal from high-dose zopiclone misuse frequently results in neurological complications, such as seizures and delirium, predominantly affecting elderly patients or those with psychiatric histories, alcohol abuse, or substance abuse. The onset of delirium and seizures post-withdrawal suggests potential neurotoxic effects of zopiclone. In our case study, the subject exhibited irritability, hallucinations, and behavioral changes post-withdrawal, which have not been previously documented. This indicates that zopiclone withdrawal may lead to more complex neuropsychiatric symptoms. Research has demonstrated significant alterations in the expression of GABA_A_α2, β1, and ϵ receptors in the lateral cerebellum of patients with schizophrenia, major depressive disorder, and bipolar disorder (31). Zopiclone intake exacerbates GABAergic dysfunction, potentially explaining the psychotic symptoms and mood alterations observed following drug withdrawal in this case.

Similar to BDZs, there is currently no standardized treatment protocol for zopiclone. Case reports regarding zolpidem dependence (32) indicate that combination pharmacotherapy may play a critical role in treatment. In this instance, the patient’s anxiety, depression, psychotic symptoms, and impulsive behavior were managed through diazepam substitution therapy in combination with trazodone hydrochloride, quetiapine fumarate, and magnesium valproate. This integrative treatment strategy not only alleviated the patient’s dependence on zopiclone but also significantly improved psychiatric symptoms and mood.

This case illustrates that exceeding the recommended dosage of zopiclone may result in dependency and severe withdrawal symptoms. Therefore, healthcare professionals should exercise caution when prescribing zopiclone, even for patients without a history of substance abuse or psychiatric disorders. Current regulatory focus on Z-drugs primarily targets zolpidem, which, according to the World Health Organization (WHO), has an abuse and dependency profile similar to BDZs. In 2001, zolpidem was classified under the same regulatory category as BDZs (33). In contrast, the prevailing view suggests that zopiclone poses a lower risk of abuse and dependency compared to zolpidem, resulting in relatively lax regulation and easier access, potentially leading to misuse. Consistent with the findings of this case, the UK’s Advisory Council on the Misuse of Drugs (ACMD) recommended in 2013 that zaleplon and zopiclone be regulated in the same manner as zolpidem (34). A 2019 study reviewing reports from the European Medicines Agency’s (EMA) suspected adverse drug reaction (ADR) database identified issues of abuse, dependency, and withdrawal associated with all Z-drugs (35). Therefore, public health policymakers should ensure that all Z-drugs, including zopiclone, are subject to appropriate regulation. This includes implementing stringent prescription management systems and limiting the duration and dosage of drug use.

## Data Availability

The raw data supporting the conclusions of this article will be made available by the authors, without undue reservation.
