# Supplementary material for: Dependence on zopiclone: a case report
Source: Front Psychiatry. 2025 May 23;16:1592065. doi: 10.3389/fpsyt.2025.1592065 (PMC12143265; doi:10.3389/fpsyt.2025.1592065)
Supplement: Supplementary file 1 [file DataSheet1.pdf]

CARE
case report guidelines

CARE Checklist of information to include when writing a case report

EY-NC-ND

Topic	Item	Checklist item description	Reported on Line
Title	1	The diagnosis or intervention of primary focus followed by the words “case report”	0....
Key Words	2	2 to 5 key words that identify diagnoses or interventions in this case report, including "case report"	10 . . .
Abstract (no references)	3a	Introduction: What is unique about this case and what does it add to the scientific literature?	17-24
	3b	Main symptoms and/or important clinical findings	17-21
	3c	The main diagnoses, therapeutic interventions, and outcomes	137-151
	3d	Conclusion—What is the main “take-away” lesson(s) from this case?	25-27
Introduction	4	One or two paragraphs summarizing why this case is unique (**may include references**)	90-95
Patient Information	5a	De-identified patient specific information	yes
	5b	Primary concerns and symptoms of the patient	97-113
	5c	Medical, family, and psycho-social history including relevant genetic information	114-119
	5d	Relevant past interventions with outcomes	107-109
Clinical Findings	6	Describe significant physical examination (PE) and important clinical findings	120-131
Timeline	7	Historical and current information from this episode of care organized as a timeline	
Diagnostic Assessment	8a	Diagnostic testing (such as PE, laboratory testing, imaging, surveys)	131-136
	8b	Diagnostic challenges (such as access to testing, financial, or cultural)	137-139
	8c	Diagnosis (including other diagnoses considered)	137-139
	8d	Prognosis (such as staging in oncology) where applicable	145-151
Therapeutic Intervention	9a	Types of therapeutic intervention (such as pharmacologic, surgical, preventive, self-care)	140-144
	9b	Administration of therapeutic intervention (such as dosage, strength, duration)	140-144
	9c	Changes in therapeutic intervention (with rationale)	145-146
Follow-up and Outcomes	10a	Clinician and patient-assessed outcomes (if available)	146-151
	10b	Important follow-up diagnostic and other test results	
	10c	Intervention adherence and tolerability (How was this assessed?)	141-151
	10d	Adverse and unanticipated events	148-149
Discussion	11a	A scientific discussion of the strengths AND limitations associated with this case report	174-176
	11b	Discussion of the relevant medical literature **with references**	153-176
	11c	The scientific rationale for any conclusions (including assessment of possible causes)	212-217
	11d	The primary “take-away” lessons of this case report (without references) in a one paragraph conclusion	225-237
Patient Perspective	12	The patient should share their perspective in one to two paragraphs on the treatment(s) they received	146-151
Informed Consent	13	Did the patient give informed consent? Please provide if requested	**Yes √ No**
